# A Knowledge Retrieval Framework for Household Objects and Actions with External Knowledge

**DOI:** 10.1007/978-3-030-59833-4_3

**Published:** 2020-10-27

**Authors:** Alexandros Vassiliades, Nick Bassiliades, Filippos Gouidis, Theodore Patkos

**Affiliations:** 8 Linköping University, Linköping, Sweden; 9 University of Amsterdam, Amsterdam, Noord-Holland The Netherlands; 10 Department of Computer Science, Vrije Universiteit Amsterdam, Amsterdam, Noord-Holland The Netherlands; 11 St. Pölten University of Applied Sciences, St. Pölten, Austria; 12 FIZ Karlsruhe – Leibniz Institute for, Karlsruhe, Germany; 13 Karlsruhe Institute of Technology, Karlsruhe, Germany; 14 UAS St. Pölten, St. Pölten, Niederösterreich Austria; 15 Vienna University of Economics and Business, Vienna, Wien Austria; 16 VU Amsterdam, Amsterdam, The Netherlands; 17 ADAPT Centre, Trinity College Dublin, Dublin, Ireland; 18 Department of Computer Science, Aristotle University of Thessaloniki, Thessaloniki, Greece; 19 Institute of Computer Science, Foundation for Research and Technology, Hellas, Heraklion, Greece

**Keywords:** Ontology, Cognitive robotics, Knowledge retrieval framework, Semantic similarity

## Abstract

In the field of domestic cognitive robotics, it is important to have a rich representation of knowledge about how household objects are related to each other and with respect to human actions. In this paper, we present a domain dependent knowledge retrieval framework for household environments which was constructed by extracting knowledge from the VirtualHome dataset (http://virtual-home.org). The framework provides knowledge about sequences of actions on how to perform human scaled tasks in a household environment, answers queries about household objects, and performs semantic matching between entities from the web knowledge graphs DBpedia, ConceptNet, and WordNet, with the ones existing in our knowledge graph. We offer a set of predefined SPARQL templates that directly address the ontology on which our knowledge retrieval framework is built, and querying capabilities through SPARQL. We evaluated our framework via two different user evaluations.

## Introduction

Ontologies have been used in many cognitive robotic systems which perform object identification [8, 22, 31], affordances detection (i.e. the functionality of an object) [2, 16, 25], and for robotic platforms that work as caretakers for people in a household environment [20, 34]. We can see an extensive survey on these topics in [9]. In this paper, we introduce a novel knowledge retrieval framework^1^ for household objects and actions that can be used as part of the knowledge representation component of a cognitive robotic system, which is connected with a custom made semantic matching algorithm to enrich its knowledge. Moreover, to the best of our knowledge our ontology is the largest one about objects and actions, as well as activities (i.e. set of object-action relations).

Common Sense (CS) knowledge is an aspect that is desired by any Artificial Intelligence (AI) system. Eventhough, there are no strict definitions on what we should consider CS knowledge. Our knowledge retrieval framework can help tackle queries that require CS reasoning, on how objects are related, and how we can perform a human scaled task. Some example queries are *“What actions can I perform with a pot?”*, or *“What other objects are related to knife, plate, and fork?”*, or even *“What can I turn on if I am in the living room?”*. Furthermore, our framework can recommend sequences of actions on how to perform a human scaled task, like *“How can I make a sandwich?”*. Our framework is based on a domain-specific ontology that we have developed which contains knowledge from the VirtualHome dataset [17, 23]. The ontology is built in OWL [19] and the Knowledge Base (KB) can be easily extended by adding new instances of objects, actions, and activities.

Due to the fact that the VirtualHome dataset covers a restricted set of objects, in order to be able to retrieve knowledge about objects on a larger scale, we developed a mechanism that can take advantage of external open knowledge bases in order to retrieve knowledge or answer queries about objects that do not exist in our KB. To this end, we have devised a semantic match making algorithm that retrieves semantically related knowledge out of three web knowledge graphs, namely DBpedia [5], ConceptNet [18], and WordNet [30]. When our framework cannot find an entity in its own KB, it uses the knowledge existing in the aforementioned KBs, to relate the unknown entity with one in our local KB. Also, the framework can provide some general knowledge about objects such as *“How much fat does a banana have?”*, with predefined SPARQL query templates addressed to DBpedia. We notice that our framework performs semantic matching only with the aforementioned ontologies.

The knowledge retrieval framework was evaluated with two different user evaluation methods. In the first one, 42 subjects were asked on how satisfied they were with the returned answers on different query categories. The results seem promising with a 82% score. While in the other evaluation, we gathered a gold standard dataset for a set of queries that our framework can answer, from a group of 5 persons not part of the first group. Then, we asked a group of 34 people to give us answers to the same queries using only information from each dataset, and we compared these with the answers of our knowledge retrieval framework.

The rest of the paper is organized as follows. In Sect. 2, we present the related work. In Sect. 3, we describe our approach and the architecture of our knowledge retrieval framework. Next, in Sect. 4 we present the results of the user evaluation. Finally, in Sect. 5 we give a discussion and the conclusion.

## Related Work

Our study balances between two fields. Firstly, our knowledge retrieval framework can be fused in a cognitive robotic system acting in a household environment. The cognitive robotic system will then enhance its knowledge about which objects are related, object properties, affordances understanding, and to semantically connect entities in its KB with entities in DBpedia, ConceptNet, WordNet. Secondly, if one considers only the ontology part of our work then this ontology would be close to other Linked Open Data KBs about products, and household objects. For the first case, we need to mention that our study can stand only as part of the knowledge representation component of a cognitive robotic system that can fill reasoning gaps.

Property extraction and creation methods, between objects in a household environment, have been implemented in many robotic platforms [8, 22, 33]. Usually an object identification is done based on the shape and the dimensions perceived by the vision module, or in some cases [2, 31] reasoning mechanisms such as grasping area segmentation, or a physics based module contribute to understand an object’s label. In [27], spatial-contextual knowledge is used to infer the label of an object, for example the object *x* is usually found near objects $y_1,\ldots ,y_n$, or *x* is found on *y*. Even though these are state of the art frameworks, the robotic platform has to extract information from two or more different ontologies, in order to link an object with an affordance.

The aspect of affordances understanding based on an ontology, mainly with OWL format, is widely studied. In [16, 25], authors try to understand affordances by observing human motion. They capture the semantics of a human movement, and correlate it with an action label. On the other hand, Jäger et al. [13] have connected objects with physical and functional properties, but the functional properties which can be considered as affordances, capture a very abstract concept, as they define only the properties *containment, support, movability, blockage*. Similarly, Beßler et al. [3] define 18 actions that can be performed on objects if some preconditions hold in each case, such as if the objects are reachable, the material of the object, among others. The affordances existing in our knowledge retrieval framework are more than 70, combined with other features. Thus, we can offer greater plurality from frameworks like the aforementioned ones.

Our study attempts to fill the gap found in the previous studies and develop a knowledge retrieval framework that would complete the missing knowledge. Our framework, compared to the previous ones can offer: (i) a predefined KB of objects related to actions, (ii) a KB with sequences of actions to achieve human scaled tasks, and (iii) a mechanism that uses semantic match making between an entity that does not exist in our KB with an entity in the KB.

Our semantic matching algorithm was mostly inspired by the works of Young et al. [35], and Icarte et al. [12] where they use CS knowledge from the web ontologies DBpedia, ConceptNet, and WordNet to find the label of unknown objects. As well as from the studies [6, 36], where the label of the room can be understood through the objects that the cognitive robotic system perceived from its vision module. One drawback that can be noticed in these works, is that all of them depend on only one ontology. Young et al. compares only the DBpedia comment boxes between the entities, Icarte et al. acquires only the property values from ConceptNet of the entities, and [6, 36] on the synonyms, hypernyms, and hyponyms of WordNet entities.

As for the second part, our study can be compared with an already existing product ontology, such as the product ontologies found in [24, 32], the more recent [28], and the general purpose ontology GoodRelations [10]. Our difference is that these ontologies offer information about objects, geometrical, physical, and material properties, and create object taxonomies and hierarchical relations. Instead, we have implemented knowledge about object affordances and we represent knowledge, about objects through their affordances. Furthermore, O-Pro [4] is an ontology for object-affordance relations, but is considerably smaller with respect to the quantity of objects and affordances. Thus, to the best of our knowledge we offer the largest ontology about object affordances, in a household environment.

## Our Approach

In this section, we describe in detail the architecture and the different aspects of our knowledge retrieval framework. In the first subsection, we describe the dataset from which we took knowledge and fused in our schema. Next, we present the ontology that is the main component of our framework. In the last subsection, we describe the algorithm that semantically matches entities from DBpedia, ConceptNet, and WordNet, with entities in our KB.

### Household Dataset

The VirtualHome dataset [17, 23] contains activities that people do at home. For each activity, there are different descriptions on how to perform them. The descriptions are present in the form of sequence of actions, i.e., steps that contain an action related with an object or objects, illustrated in Example 1. Moreover, the dataset offers a virtual environment representation for each sequence of actions with Unity^2^. The dataset contains $\sim$2800 sequences of actions, for human scaled activities. Moreover, the dataset holds more than 500 objects, usually found in a household environment, which are semantically connected with each other, and with specific human scaled actions.

#### Example 1

Browse Internet

Comment: walk to living room. look at computer. switch on computer. sit in chair. watch computer. switch off computer.


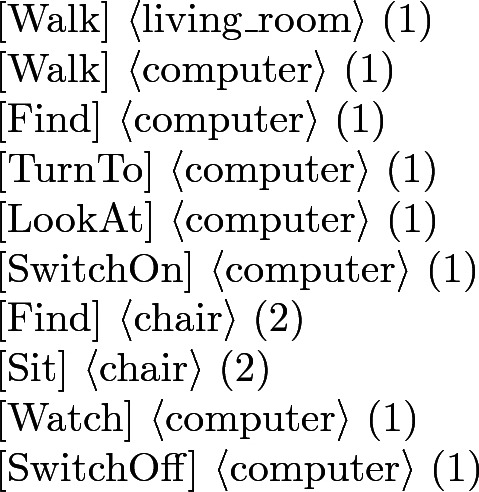


Each sequence of actions has a template: (a) Activity Label, (b) Comment, i.e. small description, and (c) the sequence of actions. Each step has the general form shown in (1):

1 $$\begin{aligned}{}[Action] \langle Object_1 \rangle (ID_1) \ldots \langle Object_n \rangle (ID_n) \end{aligned}$$

where *Action* is the human scaled action, $Object_1,\ldots ,Object_n$ are the objects on which the action is performed $\left( n \in \mathbb {N}\right)$, and $ID_1,\ldots , ID_n$ are the unique identity numbers between the objects that represent the same natural object. In our experiments we have approximately 500 objects, but due to the fact that the ontology can be freely extended with objects, we consider *n* as a natural number.

### Ontology

**Fig. 1.**
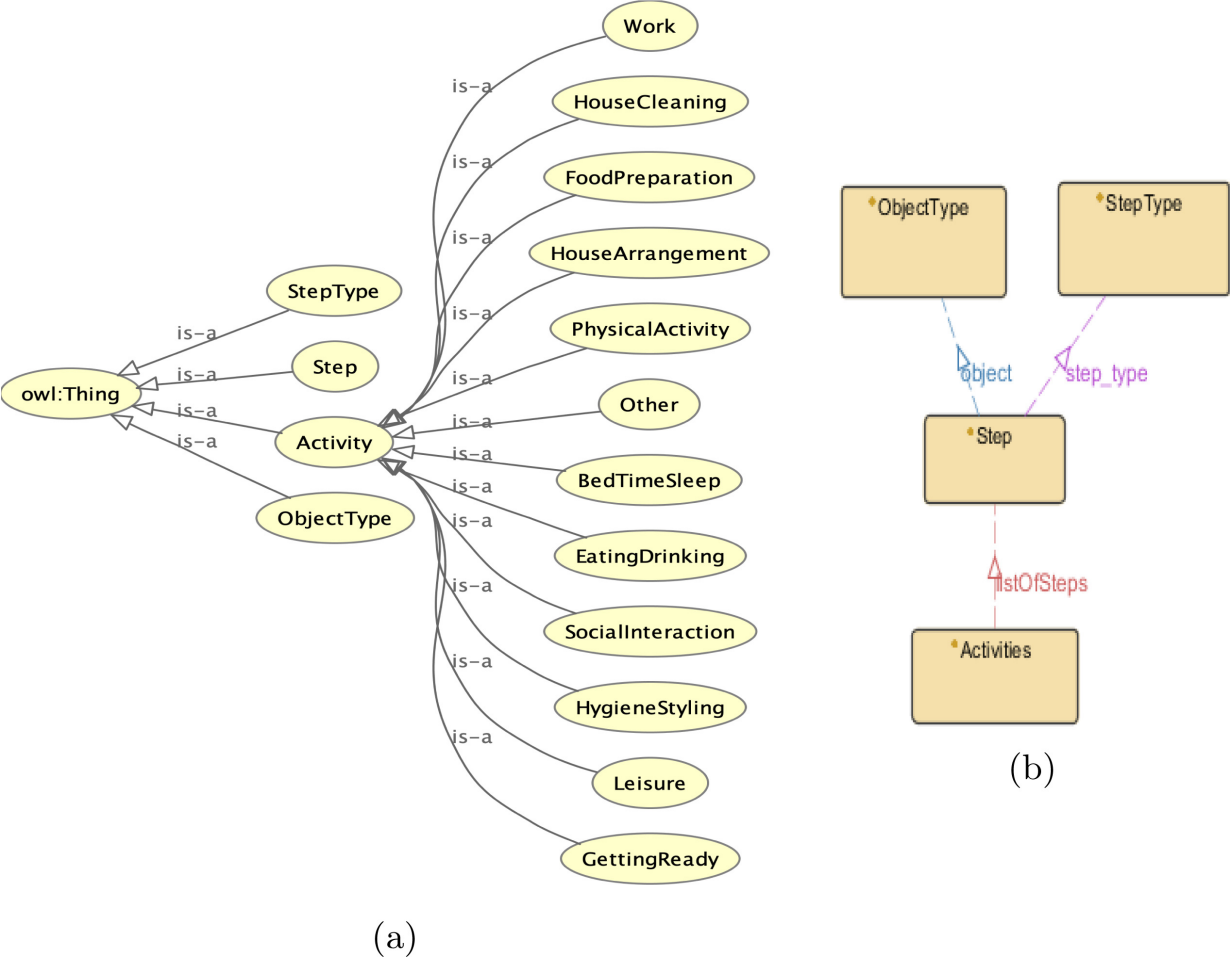
(a) Part of Ontology Scheme (b) Ontology Properties.

The main component of our knowledge retrieval framework is the ontology that was inspired by the VirtualHome dataset. Figure 1a presents part of the ontology concepts, while Fig. 1b the relationships between the major concepts.

The class *Activity* contains some subclasses which follow the hierarchy provided by the dataset; these were hand-coded. Moreover, the instances of these classes are the sequence of actions presented in the KB of the dataset. The class *Activity* is connected through the property *listOfSteps* with the class *Step*. Additionally, the class *Step* is connected through the properties *object* and *step_type* with the classes *ObjectType* and *StepType*, respectively. Next, the class *ObjectType* contains the labels of all the objects found in the sequences. On the other hand, the class *StepType* is similar to *ObjectType* as it gives natural language labels to the steps.

We have represented every sequence of actions as a list, because this gave us stronger coherency and interaction on the knowledge provided by the activity. Thus, we can answer queries like *“What is the third step in the sequence of activity X?”*, or *“Return all the sequences where firstly I walk to the living room, then I open the TV, and after that I sit on the sofa”*, information very crucial for a system with planning capabilities. Also, we have developed an instance generator algorithm that transforms the sequences of actions from the form of Example 1 into instances of classes in our ontology. The class that the sequence belongs to, is provided by the Activity label. We give such an instance in Example 2.

#### Example 2


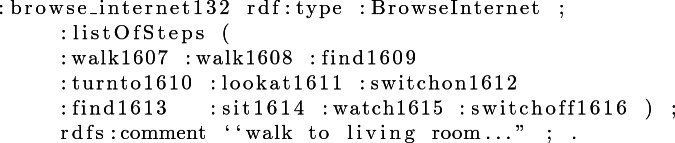


Each step shown in the property *listOfSteps* is an instance of the class *Step*. Each step has a unique ID that distinguishes it from all the other steps. Example 3 shows an instance step from the *listOfSteps*, and Example 4 the object and action with which the instance is connected from the *ObjectType* and *StepType* classes.

#### Example 3


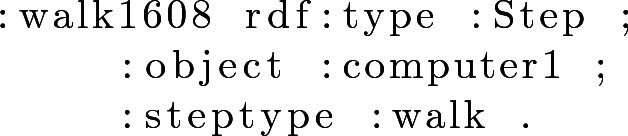


#### Example 4


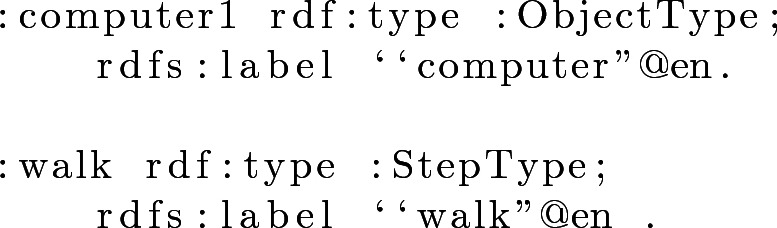


After constructing and populating the ontology, we have developed a library in Python that constructs SPARQL queries addressed to the ontology and fetches answers. The library consists of 9 predefined query templates that represent the most probable question types to the household ontology. These templates were consider as more important after an extensive literature review of studies about cognitive robotic systems that act in a household environment [9]. Among many other studies, we have considered primarily KnowRob [2, 31], RoboSherlock [1], RoboBrain [29], and RoboCSE [7]. We managed to find what were the most common and crucial queries addressed to a cognitive robotic system and we constructed these templates based on these findings. Example 5 shows the SPARQL template that returns the objects which are related to two other objects, *Object1* and *Object2*.

#### Example 5


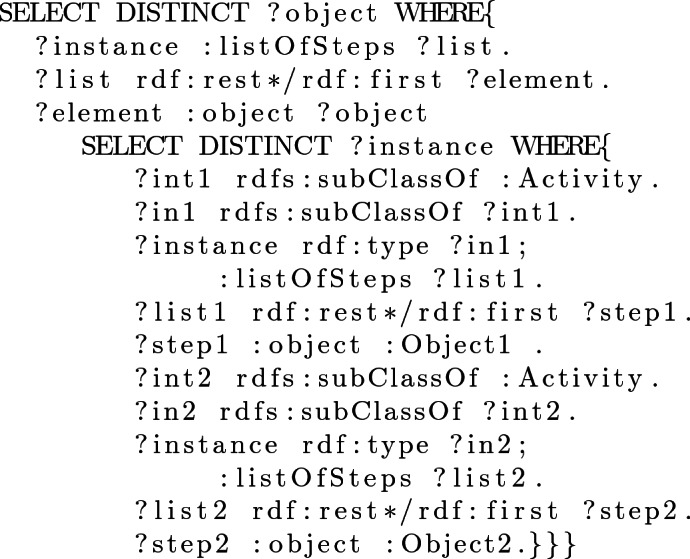


Alternatively, ad-hoc SPARQL queries can be asked to the ontology, such as Example 6 were an user wants to see the objects involved in the activity, *activity1*.

#### Example 6


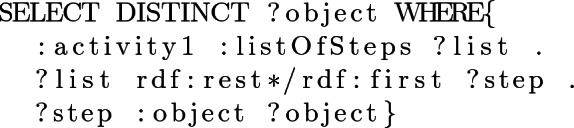


Therefore, users can hand pick one of the predefined queries and then give the keywords that are needed in order to fill the SPARQL template (Example 5), or they can write their own SPARQL query to access the information they desire (Example 6).

### Semantic Matching Algorithm

Due to the fact that the dataset upon which the knowledge retrieval framework was constructed has a finite number of objects, in order to be able to retrieve knowledge about objects on a larger scale, we developed a mechanism that can take advantage of the web knowledge graphs DBpedia, ConceptNet, and WordNet to answer queries about objects that do not exist in our KB. This would broaden the range of queries that the framework can answer, and would overcome the downside of our framework being dataset oriented. Algorithm 1 was implemented using Python. The libraries *Request* and *NLTK*^3^ offer web APIs for all three aforementioned ontologies. Similar methods can be found in [12, 35], where they also exploit the CS knowledge existing in web ontologies. Algorithm 1 starts by getting as input any word that is part of the English language; we check this by obtaining the WordNet entity, line 3. The input is given by the user implicitly, when he gives a keyword in a query that does not exist in the KB of the framework.

Subsequently, we turn to ConceptNet, and we collect the properties and values for the input word, line 4. In our framework, we collect only the values of some properties such as *RelatedTo, UsedFor, AtLocation*, and *IsA*. We choose these properties because they are the most related to our target application of providing information for household objects. Also, we acquire the weights that ConceptNet offers for each triplet. These weights represent how strong the connection is between two different entities with respect to a property in the ConceptNet graph, and are defined by the ConceptNet community. Therefore, we end up with a hash map of the following form:

$$\Big \{Property_1:\left[ \left( entity^{1}_1, weight^{1}_1\right) , \ldots ,\left( entity^{1}_m, weight^{1}_m\right) \right] ,\ldots ,$$

$$Property_l:\left[ \left( entity^{l}_1, weight^{l}_1\right) , \ldots ,\left( entity^{l}_k, weight^{l}_k\right) \right] \Big \}$$

for $$m,l,k \in \mathbb {N}\backslash \{0\}$$.

Then, we start extracting semantic similarity between the given entity and the returned property values using WordNet and DBpedia, lines 5–8. Firstly, we find the least common path that the given entity has with each returned value from ConceptNet, in WordNet, line 9. The knowledge in WordNet is in the form of a direct acyclic graph with hyponyms and hypernyms. Thus, in each case we obtain the number of steps that are needed to traverse from one entity to another. Subsequently, we turn to DBpedia to extract comment boxes of each entity using SPARQL, lines 11–13. If DBpedia does not return any results, we search the entity in Wikipedia, which has a better search engine, and with the returned URL we ask again DBpedia for the comment box, based on the mapping scheme between Wikipedia URLs and DBpedia URIs, lines 14–20. Notice that when we encounter a redirection list we acquire the first URL of the list which in most cases is the desired entity, and acquire the comment box.

The comment box of the input entity is compared with each comment box of the returned entities from ConceptNet, using the TF-IDF algorithm to extract semantic similarity, line 21. Here we follow a policy which prescribes that the descriptions of two objects which are semantically related will contain common words. We preferred TF-IDF despite its limitations, as it may miss some words only from the difference of one letter, because we did not want to raise the complexity of the framework using pre-trained embedding vectors like Glove [21], Word2Vec [26], or FastText [14], this remains as future work.
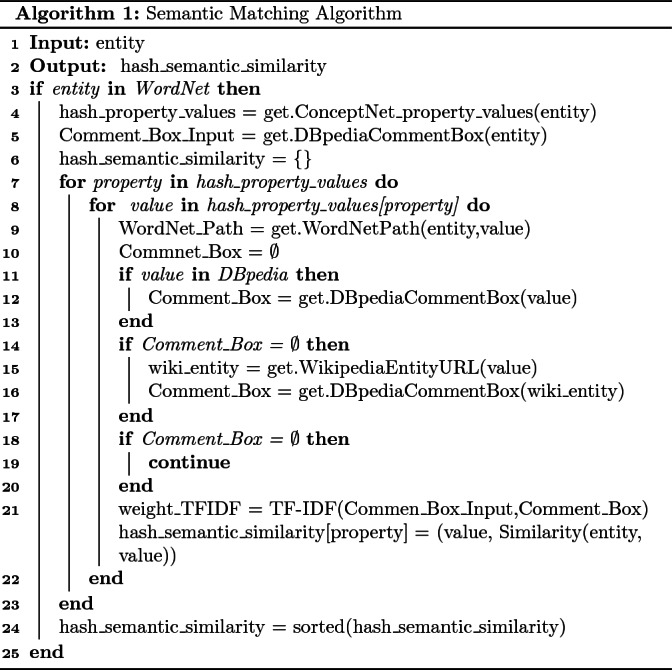


In order to define the semantic similarity between the entities, we have devised a new metric that is based on the combination of WordNet paths, TF-IDF scores, and ConceptNet weights Eq. (2). We choose this specific metric because it takes into consideration the smallest WordNet path, the ConceptNet weights, and the TF-IDF scores. TF-IDF and ConceptNet scores have a positive contribution to the semantic similarity of two words. On the other hand, the bigger the path is between two words in WordNet the smaller the semantic similarity is.

2 $$\begin{aligned} \begin{aligned} Sim(i, v) =&\frac{1}{WNP(i, v)} + TFIDF(i, v) + CNW(i, p, v) \end{aligned} \end{aligned}$$

In Eq. 2, *i* is the entity given as input by the user, and *v* is each one of the different values returned from ConceptNet properties. *CNW*(*i*, *p*, *v*) is the weight that ConceptNet gives for the triplet (*i*, *p*, *v*), and *p* stands for the property that connects *i* and *v*. *TFIDF*(*i*, *v*) is the score returned by the TF-IDF algorithm when comparing the DBpedia comment boxes of *i* and *v*. *WNP*(*i*, *v*) is a two parameter function that returns the least common path between *i* and *v*, in the WordNet direct acyclic graph.

In case *i* and *v* have at least one common hypernym (ch), then we acquire the smallest path for the two words, whereas in case *i* and *v*, do not have a common hypernym (nch), we add their depths. Let $depth(\cdot )$ be the function that returns the number of steps needed to reach from the root of WordNet to a given entity, then:

3 $$\begin{aligned} WNP(i, v) = \left\{ \begin{matrix} min_{c \in C}\left\{ depth(i) + depth(v)-2*depth(c)\right\} &{} \text {ch} \\ depth(i) + depth(v) &{}\text {nch} \end{matrix}\right. \end{aligned}$$

where *C* is the set of common hypernyms for *i* and *v*. *WNP* $(\cdot ,\cdot )$ will never be zero, as two different entities in a direct acyclic graph will always have at least one step path between them.

The last step of the algorithm sorts the semantic similarity results of the entities with respect to the ConceptNet property, and stores the new information into a hash map, line 24. An example of the returned information is given in Example 7 where the Top-5 entities for each property are displayed, if there exist as many.

#### Example 7

- coffee IsA: stimulant, beverage, acquired_taste, liquid.
- coffee AtLocation: sugar, mug, office, cafe.
- coffee RelatedTo: cappuccino, iced_coffee, irish_coffee, turkish_coffee, plant.
- coffee UsedFor: refill.

## Evaluation

We evaluated our knowledge retrieval framework via two different user evaluations. Firstly, by asking people how much they are satisfied with the results returned. Basically, we wanted to see if the answers returned by our framework satisfied the users in terms of CS. Due to the fact that we cannot define strict rules on what can be considered as CS, each subject gives their personal opinion to evaluate how satisfied they are with each answer. Thus, we asked for a score from 1 to 5 to eight categories of queries. Each person had to evaluate 40 answers (5 queries of each of the eight categories). Subjects were presented with the Top-5 answers returned for each query. We tried to find people both related to Computer Sciences (CSc) and people not related to Computer Science (N-CSc), resulting in 19 and 23 subjects, respectively. We also made another clustering with the same people based on their education level, Workers 13 (W) that did not go to University, Bachelor/Master Students 23 (B/M), PhD Students 6 (P).

The categories of queries that were evaluated are the following: Q1: *“On what objects can I perform the actions X1,..,Xn if I am in room Y?”*, Q2: *“On what objects can I perform the actions X1,..,Xn?”*, Q3: *“What can I do with objects O1,...,Om?”*, Q4: *“What objects are related to objects O1,...,Om?”*, Q5: *“Give me the category of activities for X”*, Q6: *“Give me related objects to O1,...,Om”*, Q7: *“Give me similar action(-s) to A”*, and Q8: *“Recommend an Activity based on the description* $\mathcal {A}$”. Notice that in Q4, we addressed queries with objects that do not exist in our KB, to see how satisfied people are with the recommendations from Algorithm 1. Table 1 and Table 2 present the Mean and Variance scores, respectively. The results are rounded to two decimals in all the tables.

**Table 1.** Table with Mean scores for Q1-Q8.

	General	W	B/M	P	CSc	N-CSc
Q1	4.20	4.18	4.17	4.22	4.21	4.29
Q2	4.35	4.36	4.39	4.32	4.39	4.35
Q3	4.08	4.08	4.16	4.06	4.10	4.08
Q4	3.73	3.72	3.70	3.74	3.72	3.73
Q5	4.19	4.16	4.24	4.16	4.16	4.18
Q6	4.11	4.09	4.12	4.10	4.11	4.09
Q7	3.99	4.09	3.97	3.95	3.91	4.06
Q8	4.12	4.10	4.16	4.25	4.02	4.09
Mean	4.10	4.1	4.11	4.10	4.08	4.10

**Table 2.** Table with Variance scores for Q1-Q8.

	General	W	B/M	P	CSc	N-CSc
Q1	0.96	1.52	0.95	0.78	1.20	0.74
Q2	0.80	0.83	0.76	0.92	0.71	0.87
Q3	1.12	0.5	1.44	0.91	1.25	1.06
Q4	1.52	1.06	1.52	1.69	1.51	1.51
Q5	1.61	1.54	1.59	1.65	1.73	1.49
Q6	0.98	0.86	0.95	1.09	0.97	0.98
Q7	1.75	1.75	1.82	1.38	1.88	1.66
Q8	1.56	1.46	1.54	1.74	1.64	1.52
Variance	1.20	1.13	1.21	1.21	1.26	1.13

As we can see, we obtained an overall of 4.10/5, which translates to an 82% score. Moreover, regarding the low score of Q4 in comparison to other queries we can comment the following. This happened because we had a very high threshold value to the *Ratcliff-Obershelp* string similarity metric, which compared the returned results from Algorithm 1 with the ones in our KB. On top of that, we did not display the recommendation from Algorithm 1; instead, we displayed the entity from our KB with which the result of Algorithm 1 was close enough. The threshold was 0.8 and we reduced it to 0.6; for smaller values the recommendations of Algorithm 1 in most cases were not related to our target application. Therefore, we reduced the value of the threshold and displayed the web KB recommendation. We performed these changes in order to affect only Q4. The new results are displayed in Table 3. We observe that the Mean score for Q4 increased by 13.5%, and the Variance shows that the scoring values came closer to the Mean value by 0.89.

**Table 3.** Table with Mean and Variance scores for Q4, with the new changes.

	General	W	B/M	P	CSc	N-CSc
Mean	4.41	4.35	4.36	4.6	4.45	4.36
Variance	0.61	0.74	0.65	0.39	0.42	0.73

In our second evaluation, we asked from 5 subjects not part of the first group to give us their own answers in the queries Q1-Q7, apart from Q4 (we shall denote this by Q1-Q7$\backslash$Q4). We omitted Q4 and Q8 because we consider them as less important for evaluating the capabilities of our knowledge retrieval framework. More specifically, from the viewpoint of a user Q4 is similar to Q6, so there was no point asking it again. On the other hand, for the Q8 the 5 subjects were reluctant to answer it because they considered it very time consuming (it required to provide 25 full sentences; not just words as in the case of the other queries), so we could not gather a quantitatively appropriate dataset. Therefore, the 5 subjects had to give us 5 answers based only on their own opinion for 5 queries from each one of Q1-Q7$\backslash$Q4. We resulted with a baseline dataset of 125 answers for each query. Next, 34 subjects from the first evaluation agreed to proceed with the second round of evaluation. Each one had to give one answer, for 5 queries from each one of the queries Q1-Q7$\backslash$Q4 (5 * 6 = 30 answers in total) picked from the aforementioned dataset.

4 $$\begin{aligned} Topi = \frac{{Number\ of\ correct\ answers\ in\ first\ i\ choices}}{{Number\ of\ answers\ in\ users\ category\ j}} \end{aligned}$$

where $i = 1,3,5$, and $j \in \{W, B/M, P, CSc, N-CSc \}$. Then, we compared these answers with what our knowledge retrieval framework returned to each query in the first choice (Top1), the three first choices (Top3), and in the five first choices (Top5). The results are in Table 4, and they show the precision of the system Eq. (4).

**Table 4.** Table with Top1–Top3–Top5 scores.

	General	W	B/M	P	CSc	N-CSc
Top1	71.1%	71.1%	72.9%	63.3	71.4%	71.0%
Top3	80.7%	71.1%	82.2%	75.8%	81.6%	80.1%
Top5	84.1%	82.7%	89.6%	83.3%	83.8%	84.3%

We see that we achieved a 71.1% score in the Top1 results returned by our knowledge retrieval framework, which is high if we take into consideration that this is not a data driven framework which could learn the connections between the queries and answers, nor use embeddings between queries and answers that could point to the correct answer, therefore we gave a margin of error. Hence, we also display the Top3 and Top5 choices, where we see significant improvement by 9.6% and 13%, respectively.

**Evaluation Discussion:** The evaluation unfortunately could not be done with immediate interaction with the framework, as we have not yet developed a Web API. For the first evaluation, the subjects were given spreadsheets with the queries and their answers and they had to evaluate each one of them. As for the second part, 5 subjects not part of the first group where given the queries Q1-Q7$\backslash$Q4, and they had to give their own answer, from where we collected the gold standard dataset. This procedure was done again through spreadsheets. Subsequently, 34 subjects from the first evaluation were asked to answer Q1-Q7$\backslash$Q4 using as options the words from the gold standard dataset. Therefore, the latter group were given the stack of potential answers for each query and a spreadsheet with the queries Q1-Q7$\backslash$Q4.

Considering to potential biases we notice that between the first and second evaluation there was a time lapse of over 40 d, so we doubt that any of the subjects remembered any answer from the first evaluation. Secondly, the queries were formed after an extensive literature review of what is commonly considered as crucial knowledge for cognitive robotic systems interacting with humans in a household environment. Furthermore, although we have 9 predefined SPARQL templates we have used only 8 of them in the first evaluation; this is because the one that was omitted involves the activities that were part of the VirtualHome dataset, so we have considered that this was already evaluated by previous related work.

Finally, looking at the results of the evaluation we drive the following conclusions. Firstly, the large percentage (82%) on how much satisfied with the answers of our knowledge retrieval framework the subjects are, signifies that our framework can be used by any cognitive robotic system acting in a household environment as a primary (or secondary) source of knowledge. Secondly, the second method of evaluation implies that our knowledge retrieval framework could be used as a baseline for evaluating other cognitive robotic systems acting in a household environment. Thirdly, the scores that Algorithm 1 achieved, show that it can be used as an individual service for semantically matching entities of a knowledge graph with entities from ConceptNet, DBpedia, and WordNet as it can be easily extended with more properties.

## Discussion and Conclusion

In this paper, we presented a knowledge retrieval framework that can be fused in a cognitive robotic system that acts in a household environment, and an ontology schema. More specifically, we extracted information from the VirtualHome dataset to fuse it into our framework. Furthermore, with an instance generator algorithm we translated the activities as instances of the ontology classes. Therefore, we obtained knowledge, about how actions and objects are related, what objects are related with each other, what objects and actions exist in an activity, and suggestions on how to perform an activity in a household environment, through a set of predefined SPARQL query templates. The knowledge retrieval framework can also address hand-coded SPARQL queries to its own KB. Additionally, we broadened the range of queries the framework can answer, by developing a Semantic Matching Algorithm that finds semantic similarity, between entities existing in our KB and entities from the knowledge graphs of DBpedia, ConceptNet, and WordNet.

The problem of building an ontology schema that contains a wide variety of instances and properties, is well studied [11, 15]. The same does not hold when we try to fuse CS knowledge in an KB, therefore usually methods that acquire CS either from a local KB, or a combination of local and web KBs are used. Unfortunately, fusing CS knowledge and reasoning in an ontology is not a very well-studied area, and the methods presented until now can rarely be generalized. CS knowledge and the capability of a cognitive robotic system to answer CS related queries offers flexibility.

We consider that we made a contribution in this direction by presenting a knowledge retrieval framework that can provide knowledge to a cognitive robotic system to answer questions that require CS reasoning. Looking at the results of our two evaluations we can conclude that our approach has a merit towards our aims. Firstly, the 82% score in the first evaluation where the users had to evaluate the answers based on their own CS, implies that our framework can provide knowledge for CS questions in a household environment. Additionally, the scores in the second evaluation show that the knowledge retrieval framework can be used as a baseline for evaluating other frameworks.

As for future work, we are planning to extend the scheme of the ontology with spatial information about objects, for example *soap is usually found near sink, sponge, bathtub, shower, shampoo*. Also, we plan to broaden the part of the framework which returns general knowledge about objects, by extracting knowledge from more open web knowledge graphs, in addition to DBpedia. Finally, we aim to extend the Semantic Matching Algorithm by obtaining information from other ontologies.

## Footnotes

1 https://github.com/valexande/HomeOntology.

2 https://unity.com.

3 https://www.nltk.org.

## References

[1] Beetz, M., Bálint-Benczédi, F., Blodow, N., Nyga, D., Wiedemeyer, T., Marton, Z.C.: Robosherlock: unstructured information processing for robot perception. In: 2015 IEEE International Conference on Robotics and Automation (ICRA), pp. 1549–1556. IEEE (2015)

[2] Beetz, M., Beßler, D., Haidu, A., Pomarlan, M., Bozcuoğlu, A.K., Bartels, G.: Know rob 2.0–a 2nd generation knowledge processing framework for cognition-enabled robotic agents. In: 2018 IEEE International Conference on Robotics and Automation (ICRA), pp. 512–519. IEEE (2018)

[3] Beßler, D., Koralewski, S., Beetz, M.: Knowledge representation for cognition-and learning-enabled robot manipulation. In: CogRob@ KR, pp. 11–19 (2018)

[4] Bhattacharyya R, Bhuyan Z, Hazarika SM, Basu A, Das S, Horain P, Bhattacharya S (2017). O**-**PrO: an ontology for object affordance reasoning. Intelligent Human Computer Interaction.

[5] Bizer C (2009). DBpedia-a crystallization point for the web of data. Web Semant. Sci. Serv. Agents World Wide Web.

[6] Chernova S, Amato NM, Hager G, Thomas S, Torres-Torriti M (2020). Situated Bayesian reasoning framework for robots operating in diverse everyday environments. Robotics Research.

[7] Daruna, A., Liu, W., Kira, Z., Chetnova, S.: RoboCSE: robot common sense embedding. In: 2019 International Conference on Robotics and Automation (ICRA), pp. 9777–9783. IEEE (2019)

[8] Fischer, L., et al.: Which tool to use? grounded reasoning in everyday environments with assistant robots. In: CogRob@ KR, pp. 3–10 (2018)

[9] Gouidis, F., Vassiliades, A., Patkos, T., Argyros, A., Bassiliades, N., Plexousakis, D.: A review on intelligent object perception methods combining knowledge-based reasoning and machine learning. arXiv preprint arXiv:1912.11861 (2019)

[10] Hepp M, Gangemi A, Euzenat J (2008). GoodRelations: an ontology for describing products and services offers on the web. Knowledge Engineering: Practice and Patterns.

[11] Hitzler, P., Gangemi, A., Janowicz, K.: Ontology Engineering with Ontology Design Patterns: Foundations and Applications, vol. 25. IOS Press (2016)

[12] Icarte, R.T., Baier, J.A., Ruz, C., Soto, A.: How a general-purpose commonsense ontology can improve performance of learning-based image retrieval. arXiv preprint arXiv:1705.08844 (2017)

[13] Jäger, G., Mueller, C.A., Thosar, M., Zug, S., Birk, A.: Towards robot-centric conceptual knowledge acquisition. arXiv preprint arXiv:1810.03583 (2018) 10.3389/frobt.2021.476084 PMC8082111 33937343

[14] Joulin, A., Grave, E., Bojanowski, P., Douze, M., Jégou, H., Mikolov, T.: Fasttext. zip: Compressing text classification models. arXiv preprint arXiv:1612.03651 (2016)

[15] Kendall EF, McGuinness DL (2019). Ontology engineering. Synth. Lect. Semant. Web Theory Technol..

[16] Lemaignan S, Warnier M, Sisbot EA, Clodic A, Alami R (2017). Artificial cognition for social human-robot interaction: an implementation. Artif. Intell..

[17] Liao, Y.H., Puig, X., Boben, M., Torralba, A., Fidler, S.: Synthesizing environment-aware activities via activity sketches. In: Proceedings of the IEEE Conference on Computer Vision and Pattern Recognition, pp. 6291–6299 (2019)

[18] Liu H, Singh P (2004). ConceptNet–a practical commonsense reasoning tool-kit. BT Technol. J..

[19] McGuinness DL, Van Harmelen F (2004). Owl web ontology language overview. W3C Recomm..

[20] Meditskos, G., Kontopoulos, E., Vrochidis, S., Kompatsiaris, I.: Converness: Ontology-driven conversational awareness and context understanding in multimodal dialogue systems. Expert Systems (2019)

[21] Pennington, J., Socher, R., Manning, C.: Glove: global vectors for word representation. In: Proceedings of the 2014 Conference on Empirical Methods in Natural Language Processing (EMNLP), pp. 1532–1543 (2014)

[22] Salinas Pinacho L, Wich A, Yazdani F, Beetz M, Trollmann F, Turhan A-Y (2018). Acquiring knowledge of object arrangements from human examples for household robots. KI 2018: Advances in Artificial Intelligence.

[23] Puig, X., et al.: Virtualhome: simulating household activities via programs. In: Proceedings of the IEEE Conference on Computer Vision and Pattern Recognition, pp. 8494–8502 (2018)

[24] Radinger, A., Rodriguez-Castro, B., Stolz, A., Hepp, M.: BauDataWeb: the Austrian building and construction materials market as linked data. In: Proceedings of the 9th International Conference on Semantic Systems, pp. 25–32. ACM (2013)

[25] Ramirez-Amaro K, Beetz M, Cheng G (2017). Transferring skills to humanoid robots by extracting semantic representations from observations of human activities. Artif. Intell..

[26] Rong, X.: word2vec parameter learning explained. arXiv preprint arXiv:1411.2738 (2014)

[27] Ruiz-Sarmiento JR, Galindo C, Gonzalez-Jimenez J (2015). Exploiting semantic knowledge for robot object recognition. Knowl.-Based Syst..

[28] Sanfilippo EM (2018). Feature-based product modelling: an ontological approach. Int. J. Comput. Integr. Manuf..

[29] Saxena, A., Jain, A., Sener, O., Jami, A., Misra, D.K., Koppula, H.S.: Robobrain: Large-scale knowledge engine for robots. arXiv preprint arXiv:1412.0691 (2014)

[30] Strapparava, C., Valitutti, A., et al.: Wordnet affect: an affective extension of wordnet. In: LREC, vol. 4, p. 40. CiteSeer (2004)

[31] Tenorth M, Beetz M (2017). Representations for robot knowledge in the KnowRob framework. Artif. Intell..

[32] Wagner, A., Rüppel, U.: BPO: the building product ontology for assembled products. In: Proceedings of the 7th Linked Data in Architecture and Construction workshop (LDAC 2019)’, Lisbon, Portugal (2019)

[33] Wiedemeyer, T., Bálint-Benczédi, F., Beetz, M.: Pervasive’calm’perception for autonomous robotic agents. In: Proceedings of the 2015 International Conference on Autonomous Agents and Multiagent Systems. International Foundation for Autonomous Agents and Multiagent Systems, pp. 871–879 (2015)

[34] Yang G, Wang S, Yang J (2019). Desire-driven reasoning for personal care robots. IEEE Access.

[35] Young, J., Basile, V., Kunze, L., Cabrio, E., Hawes, N.: Towards lifelong object learning by integrating situated robot perception and semantic web mining. In: Proceedings of the Twenty-second European Conference on Artificial Intelligence, pp. 1458–1466. IOS Press (2016)

[36] Young J, Blomqvist E, Hose K, Paulheim H, Ławrynowicz A, Ciravegna F, Hartig O (2017). Making sense of indoor spaces using semantic web mining and situated robot perception. The Semantic Web: ESWC 2017 Satellite Events.

